# Can targeted cover letters improve participation in health surveys? Results from a randomized controlled trial

**DOI:** 10.1186/s12874-019-0799-4

**Published:** 2019-07-17

**Authors:** Anne Illemann Christensen, Peter Lynn, Janne Schurmann Tolstrup

**Affiliations:** 1 National Institute of Public Health, University of Southern Denmark, Studiestræde 6, 1455 Copenhagen K, Denmark; 2 Institute for Social and Economic Research, University of Essex, Colchester, CO4 3SQ UK; 3 National Institute of Public Health, University of Southern Denmark, Studiestræde 6, 1455 Copenhagen K, Denmark

**Keywords:** Cover letter, Response rate, Cross sectional, Health survey, Targeted survey design

## Abstract

**Background:**

Improving response rates in epidemiologic studies is important for the generalizability of the outcome. The aim of this study was to examine whether it can be advantageous for participation to target different versions of the cover letters to different sample subgroups.

**Methods:**

A randomized trial was incorporated in a cross-sectional health survey in Denmark (*n* = 25,000) where a motivational sentence in the cover letter intended to heighten perceptions of relevance of the survey was varied among 11 sample subgroups (treatment groups). Ten different versions of a sentence outlining questionnaire themes were tested: each mentioned three out of five themes: stress, alcohol, sex, sleep problems, and contact with family and friends. An eleventh group, the control group, omitted this sentence.

**Results:**

On average, the additional motivational sentence resulted in a significantly lower response rate overall compared to the control group. However, the additional motivational sentence was found to have heterogeneous effects on survey response. Furthermore, the nature of the heterogeneity differed between the versions of the sentence. Specifically, the additional sentence tended to produce a higher response rate among the youngest age group and a lower response rate in the oldest age group compared to the generic letter. The use of *alcohol* in the motivational sentence tended to have a positive effect on response in the age group 16–24 years, and *stress* tended to have a positive effect in the age group ≥65 years. On the contrary, *sex* tended to have a negative effect in the age groups 45–64 years and ≥ 65 years. However, a significant interaction was only found between the use of stress and age group (*p* = < 0.0001).

**Conclusion:**

The findings of significant and heterogeneous effects suggest that there is potential for a targeted approach to improve both response rates and sample composition. The uneven effect of the separate themes across age groups suggests that the selection of themes to be included in the motivational sentence is important for the use of targeted appeals to be successful and warrants further research to better identify which themes works in which contexts, in which subgroups and under which circumstances.

**Trial registration:**

ClinicalTrials.gov ID: NCT03046368, retrospectively registered February 8th, 2017.

**Electronic supplementary material:**

The online version of this article (10.1186/s12874-019-0799-4) contains supplementary material, which is available to authorized users.

## Introduction

Participation rates in health surveys have been declining over the past decade, and with even steeper declines in recent years [1]. This has raised concern among epidemiologists and survey researchers who attempt to obtain estimates from population-representative samples that are generalizable to the whole population. Consequently, survey design features affecting participation have been subject to extensive research. Design features suggested to affect participation include e.g. incentives, use of pre-notification, survey administration mode, the nature of the questionnaire (e.g. contents, length, design, layout and language), and use of reminders [1–4], and the literature shows that even subtle differences in design features can affect participation [5–9].

Recently, a growing number of researchers have further experimented with ways of targeting various design features to different sample subgroups to increase participation and sample balance. Hence, in the last decade, a gradual shift has been observed from surveys in which all procedures are completely standardised to surveys in which sample subgroups are treated differently [6, 10].

Targeted design is a variant of the non-standardised approach where a targeted design feature (or set of features) that is identified in advance of field work and is not then modified during field work is applied to each subgroup in the sample [10, 11]. Hence, targeted designs require information about sample units in advance of survey collection. This information is used to identify subgroups to be treated differently, and to identify the treatment to be applied to each group. Several studies indicate that a survey that adapts personalized design features achieves higher participation rates [10]. Targeted design features known to have heterogonous effect across subgroups of sample members include e.g. the form and value of incentives [12] and length of the cover letter [13].

The effectiveness of targeted designs depends partly on the richness of information available about sample members prior to field work. For this reason they have mainly been implemented on longitudinal studies where rich information about sample members is available from previous rounds and mainly to address non-response and attrition [10]. However, a few experiments have also been performed in the cross-sectional context [14, 15]. The method has proved to be useful. However, it is not standard practice yet. Further, experiments have mainly been conducted in the contexts of social surveys, and experiments have as far as we know not been conducted in the context of health surveys. Last, even though several studies of targeted design features have been identified, evidence of the effects of targeting remains limited [10].

In view of these limitations of existing studies, we wanted to examine the potential for targeted survey design features to improve response rates and sample composition in a cross-sectional health survey in Denmark. Participation can be stimulated by either a reduction in burden or an increase in motivation [5, 6, 10]. Targeted motivational statements in the cover letters or other survey materials could be ways of improving the motivation of sample members [6]. Hence, different aspects of the survey content could be emphasised in the cover letter and target different sample subgroups with the expectation of increasing the perceived relevance and saliency of the survey. [5, 6, 16].

The first research question in this study is therefor whether cover letters with targeted content can perform better than a generic letter. The proposition is that such letters should increase the willingness of some sample members to participate, and that this will be reflected in higher response rates. However, no evidence is available about which targeted content is the most effective in a cross-sectional health surveys, so in addition to examining whether it can be advantageous for response overall to use cover letters with additional motivating content, this study also examines the effect of different content across sample subgroups in order to identify how best to target the content in future surveys. Further, given that most sample members respond anyway, sample members who are swayed by the targeted letters must have relatively low response propensities (with the generic letter). Thus, we hypothesize that targeted letters should particularly improve response in subgroups known to have low response propensity (i.e. young men, unmarried, elderly women, non-Danish background) [17]. The last research question is, therefore, whether letters can be targeted to particularly improve response rates among sample subgroups with low response propensities.

## Material and methods

A randomized trial was incorporated the Danish Health and Morbidity Survey 2017 (DHMS 2017) where a sentence in the cover letter intended to heighten perceptions of relevance of the survey was varied among sample members. DHMS 2017is based on a nationally representative random sample of 25,000 individuals (including institutionalized individuals) aged 16 years or older and resident in Denmark January 1. 2017. The sample was drawn from the adult population in Denmark using the Danish Civil Registration System. The survey was conducted between February and May 2017. Sample size was determined so it was possible to detect a 4 % point difference between each intervention group and control group. This called for a minimum of 2,000 individuals in each group.

**Fig. 1:**
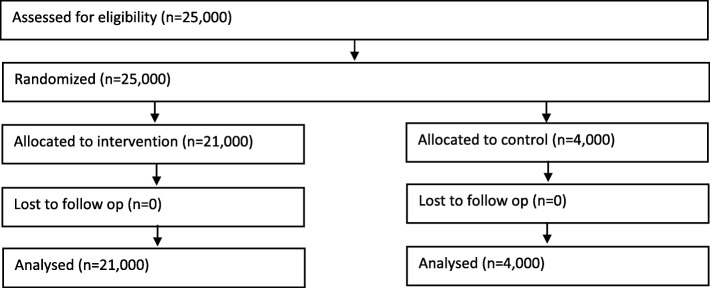
Flow diagram of the progress through the phases of a parallel randomised trial of the control and intervention groups

All sample members were randomly allocated, with equal probabilities, to one of 11 treatment groups – one control group with 4,000 individuals and 10 intervention groups with 2,100 individuals in each group. The randomization process was blinded. The control group received a generic cover letter that was designed to have general appeal and the 10 intervention groups received one of 10 versions of the cover letter including an additional motivational sentence. A flow chart is presented in Fig. 1. In the previous Health and Morbidity Surveys, all sample members received the same generic letter. Much of the content of the generic letter and the other letters was identical. The intention was to hold constant for all sample members features designed to demonstrate the credibility of the survey, to allay fears about confidentiality, to appeal to self-interest, and to provide basic information about the task of participation. All sample members were invited either to complete a web questionnaire or to fill out an identical paper questionnaire.

The additional appeal in the cover letter consisted of a sentence presenting three of five different themes in the questionnaire. The five themes were: 1) stress, 2) alcohol, 3) sex, 4) sleep problems and 5) contact with family and friends. As there is no evidence which targeted content is the most effective, the themes were selected as they were thought likely to be salient to large proportions of the population. Further, the themes were selected to represent different aspects of the survey content to examine potential heterogeneity in the appeal of the different themes between subgroups of sample members. Only three of the five themes were mentioned in each version of the letter as we wanted it to be long enough to catch attention but not so long that we would risk participants not reading the sentence fully. Further, by only mentioning three of the five themes it is possible to examine the separate effect of each theme. The sample size, the wording of the sentence, response rate overall and in the 11 treatment groups is presented in Table 1.

**Table 1:** Sample size, the wording of the additional sentence and response rate overall and in the 11 treatment groups (1 control group and 10 intervention groups)

Treatment group	Wording of the sentence	Response rate (%)	OR	95% CI	
Control group (treatment group 1/generic letter) (*n* = 4,000)	–	57.7	Ref		
Intervention group (treatment group 2/specific letter) (*n* = 2,100)	*The questionnaire will address themes such as stress, alcohol and sleep problems.*	56.8	0.96	(0.87–1.07)	*p* = 0.493
Intervention group (treatment group 3/specific letter) (*n* = 2,100)	*The questionnaire will address themes such as stress, alcohol and contact with family and friends.*	57.8	1.00	(0.90–1.12)	*P* = 0.948
Intervention group (treatment group 4/specific letter) (*n* = 2,100)	*The questionnaire will address themes such as stress, alcohol and sex*	55.6	0.92	(0.83–1.02)	*P* = 0.123
Intervention group (treatment group 5/specific letter) (*n* = 2,100)	*The questionnaire will address themes such as stress, sleep problems and contact with family and friends.*	55.8	0.93	(0.83–1.03)	*P* = 0.151
Intervention group (treatment group 6/specific letter) (*n* = 2,100)	*The questionnaire will address themes such as stress, sleep problems and sex.*	54.8	0.89	(0.80–0.99)	*P* = 0.029
Intervention group (treatment group 7/specific letter) (*n* = 2,100)	*The questionnaire will address themes such as stress, sex and contact with family and friends.*	56.5	0.95	(0.86–1.06)	*p* = 0.368
Intervention group (treatment group 8/specific letter) (*n* = 2,100)	*The questionnaire will address themes such as sex, sleep problems and contact with family and friends.*	53.4	0.84	(0.76–0.93)	*p* = 0.001
Intervention group (treatment group 9/specific letter) (*n* = 2,100)	*The questionnaire will address themes such as sex, alcohol and contact with family and friends.*	54.8	0.89	(0.80–0.99)	*p* = 0.032
Intervention group (treatment group 10/specific letter) (*n* = 2,100)	*The questionnaire will address themes such as sex, sleep problems and alcohol.*	55.8	0.93	(0.83–1.03)	*P* = 0.161
Intervention group (treatment group 11/specific letter) (*n* = 2,100)	*The questionnaire will address themes such as alcohol, sleep problems and contact with family and friends.*	56.7	0.96	(0.86–1.07)	*P* = 0.471

All sentences were introduced with the sentence: ‘You have been randomly selected to participate in a survey of well-being, health and disease among adolescents and adults in Denmark’. Percent (descriptive) and OR (logistic regression)

### Statistics

The analysis is based on descriptive statistics and logistic regression modelling of the 25,000 persons invited to DHMS 2017. In all analyses, the dependent variable indicates whether the sample member has fully or partially completed the questionnaire. In the first step, the independent variable is a dichotomous indicator of treatment (additional sentence vs. generic letter). This analysis will give an initial indication of whether, on average, the additional sentence has any effect on response propensity (Table 1). In the second step, we aim to investigate whether any such effect is heterogenous across subgroups (i.e. significantly mediated by the mediator variables). Mediator variables are four socio-demographic characteristics known to be associated with survey response propensity (sex, age, marital status, ethnic background) (Table 2). This should identify heterogeneity of effects between letter versions within sample subgroups (i.e. how best to target the letters to subgroups). Last, we investigate the main effect of each theme, by combining the intervention groups in five different ways to produce five dichotomies, each indicating whether the sentence in the cover letter included the specific theme or not (Table 3). All analysis was carried out using SAS version 9.4.

**Table 2:** Response rate overall by sex, age and marital status. Percent (descriptive) and OR (logistic regression)

	Control group (treatment group 1/generic letter) (%)	N (Treatment group 1)	Intervention groups (treatment group 2–11/specific letter) (%)	N (Treatment group 2–11)	OR (specific vs. generic)	95% CI	
Total	57.7	4000	55.8	21000	0.93	(0.87–0.99)	P = 0.028
Sex							
Men	52.6	1993	51.4	10449	0.95	(0.86–1.05)	*p* = 0.302
Women	62.7	2007	60.2	10551	0.90	(0.82–0.99)	*p* = 0.034
Age							
16–24 years	42.6	566	46.6	2975	1.18	(0.98–1.41)	*p* = 0.077
25–44 years	49.3	1150	47.4	6187	0.93	(0.82–1.05)	*p* = 0.232
45–64 years	64.3	1350	61.3	6856	0.88	(0.78–0.99)	*p* = 0.037
≥65 years	67.6	934	64.1	4982	0.86	(0.74–1.00)	*p* = 0.044
Men							
16–24 years	35.9	281	40.2	1529	1.20	(0.92–1.56)	*P* = 0.178
25–44 years	41.8	588	40.7	3195	0.95	(0.80–1.14)	*P* = 0.592
45–64 years	57.9	707	56.9	3454	0.96	(0.82–1.13)	*P* = 0.639
≥65 years	70.3	417	65.6	2271	0.81	(0.64–1.01)	*P* = 0.063
Women							
16–24 years	49.1	285	53.4	1446	1.19	(0.92–1.53)	*P* = 0.187
25–44 years	57.1	562	54.6	2992	0.90	(0.75–1.08)	*P* = 0.267
45–64 years	71.4	643	65.7	3402	0.77	(0.64–0.93)	*P* = 0.005
≥65 years	65.4	517	62.9	2711	0.90	(0.74–1.10)	*P* = 0.290
Marital status							
Married	67.9	1847	63.7	9715	0.83	(0.75–0.92)	P = 0.001
Divorced	56.5	1448	54.5	7690	0.92	(0.75–1.13)	*P* = 0.441
Widowed	54.1	439	55.2	2305	1.04	(0.80–1.36)	*p* = 0.752
Unmarried	45.7	266	46.3	1290	1.03	(0.92–1.15)	*P* = 0.665
Ethnic background							
Danish	61.1	3480	58.5	18442	0.90	(0.83–0.97)	P = 0.005
Western	38.1	205	41.3	991	1.14	(0.84–1.56)	*P* = 0.393
Non-western	33.0	315	33.2	1597	1.01	(0.78–1.30)	*P* = 0.954

**Table 3:** OR of response (inclusion of the specific theme in the motivational sentence vs. not) by age group. OR (logistic regression)

	16–24 years	95% CI		25–44 years	95% CI		45–64 years	95% CI		≥65 years	95% CI		All	95% CI	
Stress	0.97	(0.84–1.13)	*p* = 0.707	0.97	(0.88–1.08)	*p* = 0.617	1.03	(0.94–1.14)	*P* = 0.503	1.21	(1.07–1.36)	*P* = 0.002	1.04	(0.99–1.10)	*P* = 0.148
Alcohol	1.12	(0.96–1.29)	*p* = 0.147	1.07	(0.97–1.19)	*p* = 0.181	1.06	(0.96–1.17)	*P* = 0.228	0.96	(0.85–1.08)	*P* = 0.448	1.05	(0.99–1.11)	*P* = 0.100
Sex	0.98	(0.89–1.14)	*p* = 0.826	1.01	(0.91–1.12)	*p* = 0.827	0.90	(0.82–1.00)	*P* = 0.046	0.86	(0.77–0.97)	*P* = 0.015	0.94	(0.89–0.99)	*P* = 0.022
Sleep problems	0.93	(0.80–1.08)	*p* = 0.331	0.97	(0.87–1.07)	*p* = 0.521	0.99	(0.90–1.10)	*P* = 0.913	0.98	(0.87–1.10)	*P* = 0.695	0.98	(0.92–1.03)	*P* = 0.364
Contact with family and friends	1.01	(0.87–1.17)	*p* = 0.908	0.98	(0.88–1.08)	*p* = 0.677	1.01	(0.92–1.12)	*P* = 0.816	1.03	(0.91–1.16)	*P* = 0.646	1.00	(0.95–1.06)	P= 0.910

## Results

**Fig. 2:**
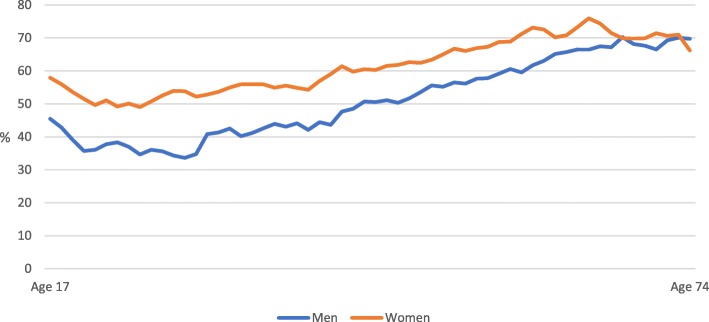
Response rate by running mean age (± 2 years). Percent

The overall response rate was 56.1%. The response rate was lower among men than women in all age groups with exception of the oldest age group (Fig. 2). The response rate was lowest in the youngest and the oldest age groups among both men and women.

On average, the letters with the additional motivational sentence (intervention group) produced an overall lower response rate (55.8%) compared to the generic letter (control group) (57.7%) (OR: 0.93; 95CI: 0.87–0.99; *p* = 0.028) (Table 2). However, differences in response were observed between subgroups of sample members defined by the mediator variables (Table 2). The letters with the additional motivational sentences (intervention group) tended to produce an overall higher response rate among the youngest age group (16–24 years), and a lower response rate in the older age groups (≥45 years) compared to the generic letter (control group). Hence, a significant interaction was found between age group and treatment group (intervention vs. control) (*p* = < 0.0001). Further, a significant interaction was observed between marital status, which is very age-dependent, and treatment group (intervention vs. control) (*p* = < 0.0001). No significant interaction was detected between sex and treatment group (intervention vs. control) (*p* = < 0.42) or between ethnic background and treatment group (intervention vs. control) (*p* = < 0.25).

The response rate varied by sex and age in the 11 treatments groups (Additional file 1: Table S1). E.g. the letter with the additional sentence ‘*stress, alcohol and contact with family and friends’* seemed to produce a higher response rate among young men (16–24 years) compared to the generic letter (control group), and among young women the letters with the additional sentence ‘*stress, sex and contact with family and friends’* and *‘sex, sleep problems and alcohol’* seemed to produce a higher response rate compared to the generic letter (control group). Further, the letter with the additional sentence ‘*sex, sleep problems and alcohol’* seemed to produce a lower response rate among women age 45–64 years compared to the generic letter (control group). However, no significant difference was observed.

The separate effect of the included themes differed by age group. The use of *alcohol* in the additional sentence tended to have a positive effect on response in the youngest age group (16–24 years), and *stress* tended to have a positive effect in the oldest age group (≥65 years) (Table 3). On the contrary, *sex* tended to have a negative effect in the age groups 45–64 years and ≥ 65 years. However, a significant interaction was only found between the use of stress and age group (*p* = < 0.0001).

## Discussion

In this randomised trial, we examined the use of specific appeals for participation in health surveys. Cover letters with an additional sentence (intervention group) intended to increase response-motivation resulted, on average, in a significantly lower response rate overall (55.8%) compared to the generic letter (control group) (57.7%). The specific letters (intervention group) tended, on average, to produce a higher response rate among the youngest age group (16–24 years) and a lower response rate in the older age groups (≥45 years). The inclusion of *alcohol* in the additional sentence tended to have a positive effect on response in the youngest age group and the inclusion of *stress* tended to have a positive effect in the oldest age group. However, *sex* tended to have a negative effect in the oldest age groups.

A previous study nested in a panel survey showed that a targeted cover letter can increase response rate, but that effects are uneven across survey design context and sample subgroups [6]. The positive effects were only observed among relatively low response propensity subgroups (previous wave non-respondents and relatively recent panel entrants). Further, differences were observed with respect to data-collection mode). This is partly in line with the findings in the present study where a positive effect was mainly observed among the younger and older age groups. This suggests that targeting should be able to improve both response rate and sample composition. Notably, with the generic letter the response rate was 35.9% among men and 49.1% among women age 16–24 whereas the specific letters provided a response rate of 40.2 and 53.4% on average, respectively. Further, the uneven effect of the separate themes across age groups suggests that the selection of themes to be included in the motivational sentence is important for the use of targeted appeals to be successful and warrants further research to better identify which themes works in which contexts and for which subgroups to best target the messaging. The heterogeneity of effects over randomized design features (mode of data-collection and time in panel) observed in the previous study suggest that the survey design context matters and that targeting might not be equally effective in all contexts. However, it was not possible to examine this in the present study.

Some might argue that post-hoc weighting adjustments could achieve the same potential benefits of targeted features with respect to handling nonresponse. Variables used to define targeted groups could as well be used as weighting variables. However, improvements in precision cannot be achieved through weighting. Further, weighting don’t have the potential to reduce any component of nonresponse bias that arises within weighting classes rather than between classes. In both cases, improvements can potentially be achieved with targeted designs. Further, non-response bias could be reduced to a greater extent than would be possible with weighting if sample members within a targeted group who participate with a targeted feature but would not have done so in the absence of the targeted feature are systematically different from other respondents in the group [18].

The study benefitted from several strengths. First, the incorporation of a randomized trial in a national representative cross-sectional health survey made it possible to study the effect of cover letters with differing appeal messages in a real-life setting based on a highly scientific method. This study thereby adds to the scientific epidemiological evidence about a relevant topic in health survey methodology, which, so far, has mainly been studied in the context of longitudinal social surveys. Although the study was conducted with great care, it had some methodological limitations. First, information about which themes motivated participation was not available and themes were therefore selected claiming they were likely to be of broad interest. This may not be the case and further studies should elaborate on the present findings to assess which themes work in which contexts, in which subgroups and under which circumstances. Further, multiple comparison was conducted in the study and some significant associations may have occurred even if there were no real differences. However, this study provides a solid knowledge base which can used as an important stepping-stone for future studies. Another limitation concerns the sample size. Many of the observed tendencies failed to reach conventional levels of significance. This might be explained by lack of power, though the study has a relatively large sample size overall (*n* = 25.000). Overall, the specific targeted designs that are suggested by the findings of this exploratory study should be tested in future confirmatory studies.

## Conclusion

On average, cover letters with an additional sentence intended to increase response-motivation resulted in a significantly lower response rate overall compared to a generic letter (control group). However, the specific letters tended to produce a higher response rate among the youngest age group and a lower response rate among the oldest age groups compared to the generic letter (control group). Furthermore, the effect of a specific letter depended on the content of the letter: the separate effects of the themes included in the motivational sentence differed by age. The inclusion of *alcohol* in the motivational sentence tended to have a positive effect on response in the youngest age group and the inclusion of *stress* tended to have a positive effect in the oldest age group. However, *sex* tended to have a negative effect in the oldest age groups.

Further research is warranted to examine which themes works in which contexts, in which subgroups and under which circumstances. This study provides a solid evidence base and an important step-stone for future studies on the context of health survey methodology. However, it is clear already that targeted letters have the potential to improve response rate and sample composition in a general population health survey of this kind. For example, consider a targeted design that would involve sending a cover letter including the motivational sentence with the themes *‘stress, alcohol, contact with family and friends’* to men aged 16–24-year-old, and *‘stress, sex, contact with family and friends’* to women aged 16–24-year-old and men aged 25–44-year-old, and the generic letter (control group) to all other subgroups. Our results suggest that the overall survey response rate would be 59.8% with the targeted design instead of 57.6% if the generic letter (control group) were sent to the whole sample. Sample composition would also be improved, with 13.0% of respondents being in the 16–24 age group, compared to 10.4% if the generic letter (control group) alone were used (14.2% is the proportion of the gross sample in this age group).

## Additional file

Additional file 1: **Table S1.** Response rate in the 11 treatment groups (1 control group and 10 intervention groups) by age. Percent (descriptive). (DOCX 18 kb)

## Data Availability

The datasets used and/or analysed during the current study are available from the corresponding author on reasonable request.

## Abbreviations

CI: Confidence intervals
DHMS 2017: The Danish Health and Morbidity Survey 2017
OR: Odds ratio
