# Supplementary material for: Can targeted cover letters improve participation in health surveys? Results from a randomized controlled trial
Source: BMC Med Res Methodol. 2019 Jul 17;19:151. doi: 10.1186/s12874-019-0799-4 (PMC6635988; doi:10.1186/s12874-019-0799-4)
Supplement: Supplementary file 1 — Table S1. Response rate in the 11 treatment groups (1 control group and 10 intervention groups) by age. Percent (descriptive). (DOCX 18 kb) [file 12874_2019_799_MOESM1_ESM.docx]

Additional file 1: **Table S1.** *Response rate in the 11 treatment groups (1 control group and 10 intervention groups) by age. Percent (descriptive)*

|  | **Men** | | | | |  | **Women** | | | | |
| --- | --- | --- | --- | --- | --- | --- | --- | --- | --- | --- | --- |
|  | **16-24 years** | **25-44 years** | **45-64 years** | **≥65 years** | **P for trend** |  | **16-24 years** | **25-44 years** | **45-64 years** | **≥65 years** | **P for trend** |
| Control group (treatment group 1/generic letter) | 35.9 | 41.8 | 57.9 | 70.3 | *P˂0.0001* |  | 49.1 | 57.1 | 71.4 | 65.4 | *P˂0.0001* |
| Intervention group (treatment group 2 - stress, alcohol and sleep problems) | 42.6 | 40.2 | 58.8 | 69.7 | *P˂0.0001* |  | 55.1 | 51.5 | 66.1 | 66.0 | *P=0.0003* |
| Intervention group (treatment group 3 - stress, alcohol and contact with family and friends) | 49.3 | 41.1 | 60.1 | 65.9 | *P˂0.0001* |  | 53.1 | 57.0 | 69.0 | 62.2 | *P=0.009* |
| Intervention group (treatment group 4 - stress, alcohol and sex) | 38.2 | 39.1 | 53.4 | 68.1 | *P˂0.0001* |  | 52.7 | 57.0 | 66.4 | 64.0 | *P=0.005* |
| Intervention group (treatment group 5 - stress, sleep problems and contact with family and friends) | 38.7 | 40.1 | 56.5 | 71.6 | *P˂0.0001* |  | 43.8 | 51.8 | 67.2 | 66.3 | *P˂0.0001* |
| Intervention group (treatment group 6 - stress, sleep problems and sex) | 43.5 | 41.6 | 57.3 | 67.3 | *P˂0.0001* |  | 46.9 | 54.1 | 63.2 | 58.6 | *P=0.006* |
| Intervention group (treatment group 7 - stress, sex and contact with family and friends) | 35.8 | 44.3 | 57.5 | 66.8 | *P˂0.0001* |  | 61.1 | 51.2 | 64.1 | 66.8 | *P=0.005* |
| Intervention group (treatment group 8 - sex, sleep problems and contact with family and friends) | 40.1 | 35.5 | 53.4 | 57.6 | *P˂0.0001* |  | 53.5 | 53.3 | 64.8 | 64.6 | *P=0.001* |
| Intervention group (treatment group 9 - sex, sleep problems and alcohol) | 37.0 | 40.1 | 56.9 | 60.1 | *P˂0.0001* |  | 58.1 | 56.9 | 62.9 | 61.8 | *P=0.195* |
| Intervention group (treatment group 10 - sex, alcohol and contact with family and friends) | 38.8 | 40.8 | 58.1 | 65.4 | *P˂0.0001* |  | 56.8 | 58.1 | 65.9 | 54.0 | *P=0.867* |
| Intervention group (treatment group 11 - alcohol, sleep problem and contact with family and friends) | 39.1 | 43.5 | 56.9 | 63.7 | *P˂0.0001* |  | 55.0 | 55.0 | 67.5 | 64.9 | *P=0.003* |
